# GIANT CUTANEOUS HORN

**DOI:** 10.4103/0019-5154.44800

**Published:** 2008

**Authors:** M Kumaresan, Pramod Kumar, Manohar Varadharaj Pai

**Affiliations:** *From the Department of Dermatology and STD, Kasturba Medical College, Mangalore, India*

**Keywords:** *Cutaneous horn*, *giant*, *seborrheic keratosis*

## Abstract

A 53-year-old male presented with a giant cutaneous horn over the left leg. Cutaneous horn was excised and primary closure of the defect was done under spinal anesthesia. Histopathology showed underlying seborrheic keratosis. Cutaneous horn has been noticed on top of many clinical conditions of diverse etiology, such as actinic keratoses, wart, molluscum contagiosum, seborrheic keratoses, keratoacanthoma, basal cell and squamous cell carcinoma. We report a patient with giant cutaneous horn on the leg successfully treated by excision and wound closure.

## Introduction

Cutaneous horn has been noticed on top of many clinical conditions like actinic keratosis, wart moluscum contagiosum, seborrheic keratoses, keratoacanthoma, basal cell carcinoma and squamous cell carcinoma. We report a case of giant cutaneous horn overlying a seborrheic keratosis treated with surgical excision and primary closure.

## Case Report

**Fig. 1:**
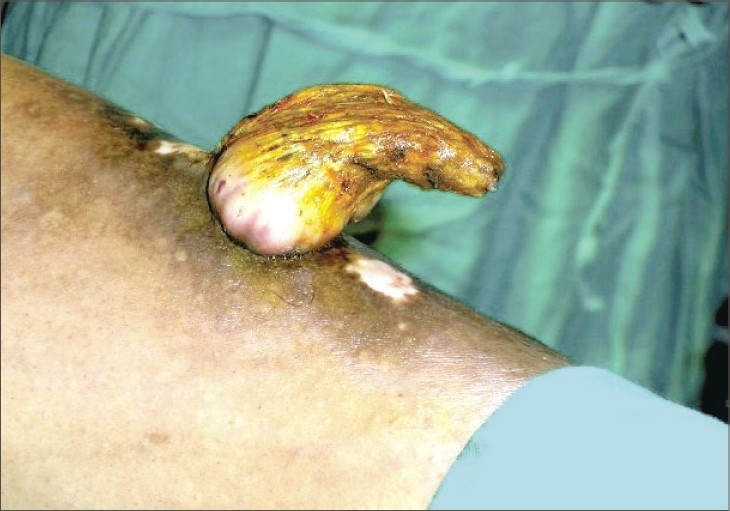
Giant cutaneous horn

**Fig. 2:**
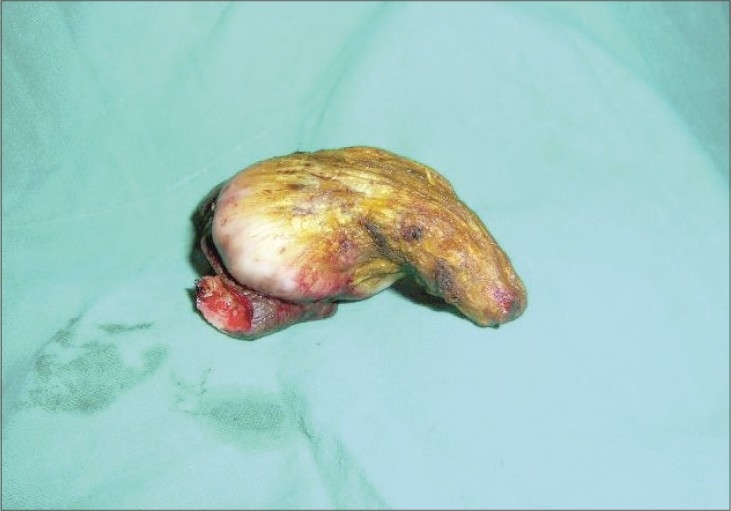
Excision of cutaneous horn

**Fig. 3:**
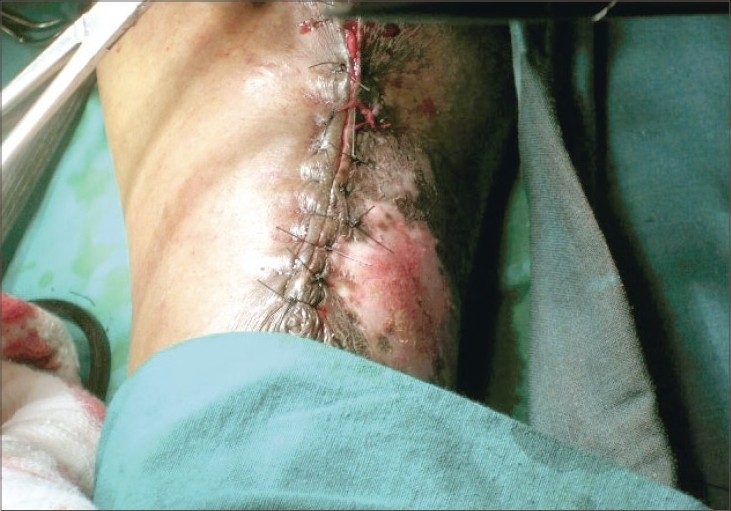
Primary closure of wound

**Fig. 4:**
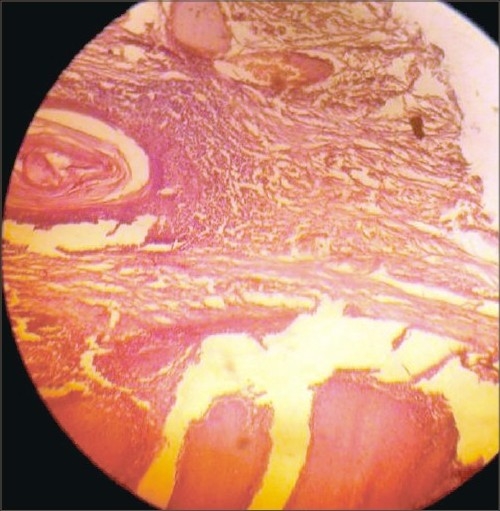
Histopathology – haematoxylin and eosin stain (40 ×)

A 53-year-old male presented with a horny projection on the left leg of 8 months duration. Initially, he had itchy hyperpigmented plaques on the leg of 4-year duration and the horny growth developed gradually over the plaque. There was no history of pain or discharge over the growth. On examination, there was a hyperkeratotic growth of 6 × 3 cm size (Fig.1) arising over a lichenified plaque on the anterior aspect of the left leg. There was no tenderness or bleeding from the growth. There were multiple lichenified plaques over the anterior aspect of the left leg. There was no regional lymphadenopathy. A clinical diagnosis of cutaneous horn overlying hypertrophic lichen planus was made. Excision of the cutaneous horn with an elliptical incision and primary closure of defect was done under spinal anesthesia (Figs. 2 and 3). Histopathological examination revealed hyperplastic skin with hyperkeratosis and parakeratosis. The epidermis showed irregular acanthosis, elongated rete ridges and papillomatosis and horn cyst. There was sparse chronic inflammatory cells infiltration in the dermis. Features were suggestive of cutaneous horn overlying a seborrheic keratosis (Fig. 4), in contrast to the clinical diagnosis of hypertrophic lichen planus

## Discussion

Cutaneous horns are elongated, keratinous projections from the skin, ranging in size from a few millimeters to many centimeters that resembles a miniature horn. The base of the horn may be flat, nodular or crateriform. The horn is composed of compacted keratin. The distribution of cutaneous horns usually is in sun-exposed areas, particularly the face, pinna, nose, forearms and dorsal hands. Usually, a cutaneous horn is several millimeters long. Malignancy is present in 16–20% of cases, with squamous cell carcinoma being the most common type.^1^ Tenderness at the base of the lesion and lesions of larger size favor malignancy. Most cutaneous horns arise from actinic keratoses but they may also result from seborrheic keratoses, warts, keratoacanthomas, squamous cell carcinomas and basal cell carcinomas. Histologically, there is a greatly thickened stratum corneum with scattered areas of parakeratosis. The horn at the base will display features characteristic of the pathologic process responsible for the development of the horn.^2^,^3^

Excision biopsy of the lesion and histopathological examination to rule out malignancy is recommended. Malignancies should be excised with appropriate margins and evaluated for metastasis. A careful physical examination of the lymph nodes draining the area of lesion often is adequate. Local destruction with cryosurgery is first-line treatment for verruca vulgaris, actinic keratosis and molluscum contagiosum.

Treatment options include wide surgical excision with careful histological examination to exclude a focus of malignancy and carbon dioxide or Neodymium YAG laser is used for patients who refuse surgery.^4^
